# Preliminary Evaluation of lutein and zeaxanthin variability in DDGS from corn ethanol facilities

**DOI:** 10.1186/s13068-025-02709-3

**Published:** 2025-11-27

**Authors:** Emily Aicher, Abigail S. Engelberth

**Affiliations:** 1 Department of Agricultural and Biological Engineering, Purdue University, 225 S. University Street, West Lafayette, IN 47907 USA; 2 Laboratory of Renewable Resources Engineering, Purdue University, 225 S. University Street, West Lafayette, IN 47907 USA

**Keywords:** Bioethanol, Lutein, Zeaxanthin, Quality, DDGS

## Abstract

Navigating the unpredictable nature of feedstock is crucial in bioprocessing. Manufacturing designs for homogeneous inputs to produce high-quality outputs, but biomass is inherently inconsistent. In bioethanol production, ethanol yield can depend on the type of corn itself in addition to the processing conditions to which it is subjected. While bioethanol processing adheres to standard operating conditions, there remains variability between facilities, an important point when considering the bioethanol byproduct DDGS as a commercial source for lutein and zeaxanthin extraction. Lutein and zeaxanthin are derived commercially from marigold flowers, which is chemically intensive and prohibits new players from entering the market. Exploring the potential of DDGS as a source of lutein and zeaxanthin would incentivize both corn growers and biorefineries but requires characterizing the differences in the yield potential of DDGS from separate bioethanol facilities. In this study, defatted DDGS from five Midwestern bioethanol facilities were examined using Soxhlet extraction with ethanol and subsequent HPLC analysis for lutein and zeaxanthin content. Using nonparametric methods and α = 0.05, it was determined that although facility appeared to have a significant effect on zeaxanthin yield, lutein yield displayed a greater degree of variability and differences in zeaxanthin yield could not be statistically ascribed to facility. Ultimately, the variability in corn growth and harvest and the downstream processing are too great to fully attribute any differences to facility alone. Future studies should consider sampling intermediate products throughout the biorefining process, as well as analyzing the incoming corn directly and comparing it to the final defatted DDGS to more fully understand how lutein and zeaxanthin partition and/or degrade during processing for a single variety of corn.

**Graphical Abstract:**

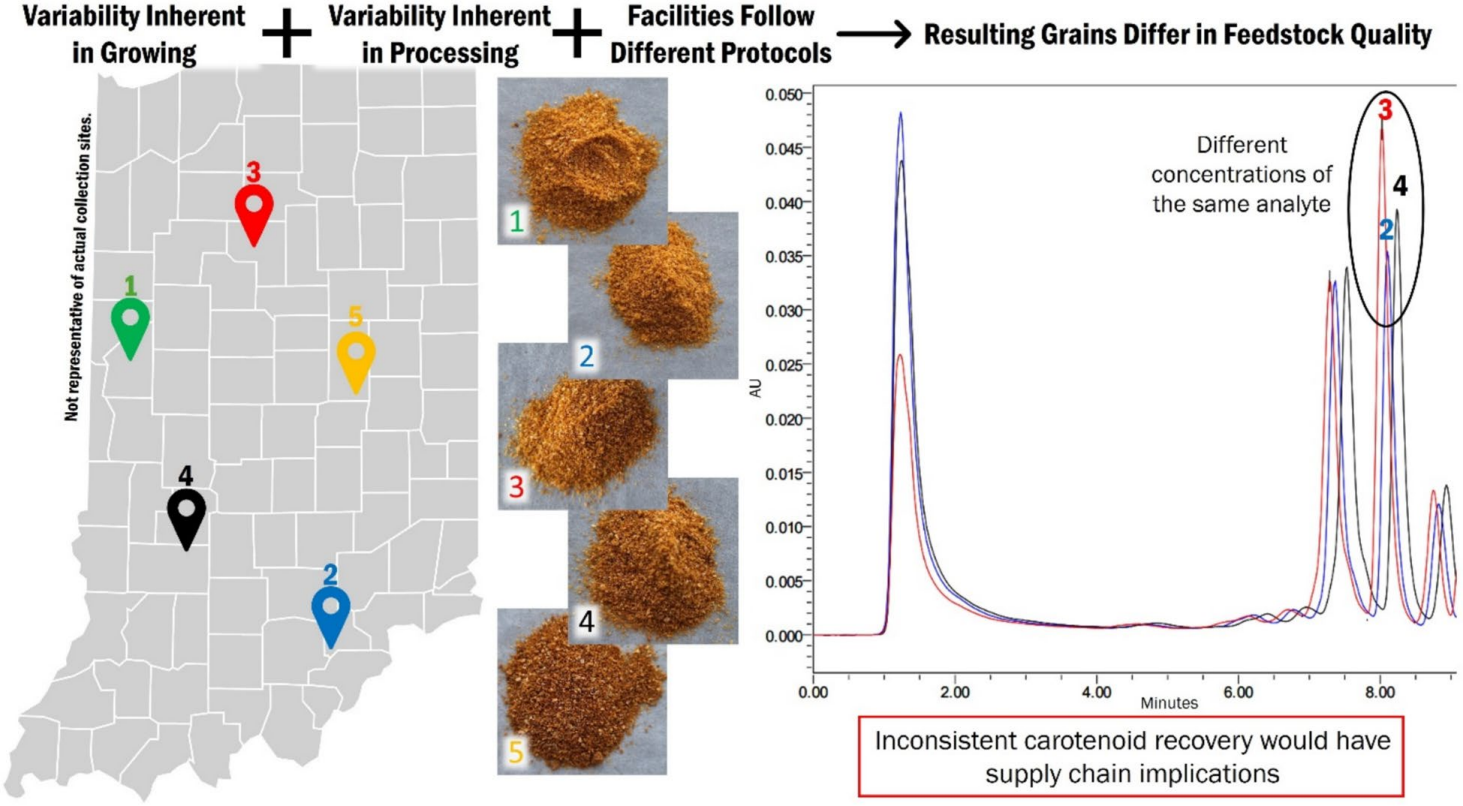

## Background

The growing demand for renewable fuels has contributed to an increase in production and investment in United States bioethanol production [1]. Corn is grown nationwide and processed to maximize bioethanol yield while also utilizing byproducts and process water to minimize waste. However, corn is not a homogeneous entity. Across the United States, hundreds of corn hybrids are grown and developed, typically designed to maximize yield or acquire traits that eliminate growth and harvest challenges [2]. Corn is also a known source of lutein and zeaxanthin, dietary supplements that contribute to the health of eye tissue [3]. Commercial lutein and zeaxanthin sources are limited, but corn and corn byproducts may have untapped potential in the supplement market [3, 4]. Specifically, DDGS as a byproduct of bioethanol production has been shown to concentrate lutein and zeaxanthin when compared to unprocessed corn [5].

Sustainability in bioethanol production continues to improve, working to recover greater degrees of water, energy, and byproducts. In addition to process water recirculation, CO_2_ can be captured and utilized for dry ice, and beverage bottling, among others [6]. Individual aspects of the refining process have been designed to increase yield or reduce energy inputs, and focus has turned to include valorization of other byproducts such as distiller’s grains. Specifically, bioethanol refining from corn follows several basic steps: milling, liquefaction, fermentation, distillation, and dehydration [7]. After distillation, the whole stillage (non-ethanol fermentation dregs) is centrifuged, producing thin stillage and wet cake (distiller’s grains, or DG) [7]. Thin stillage is further evaporated into condensed distiller’s solubles (CDS), which is mixed with wet cake to produce wet distiller’s grains and subsequently wet distiller’s grains and solubles (WDGS) [7]. Once dried, dried distiller’s grains and solubles, DDGS, are produced and typically sold as animal feed due to its high nutrient content, specifically protein [8]. Importantly, the high fat content in DDGS impacts milk quality in dairy cows and meat quality in swine, so many facilities utilize an oil recovery step from thin stillage to further valorize their DDGS [1].

Due to the vast amount of research pertaining to biorefining, modern bioethanol plants have a vast array of outputs stemming from coproducts generated from numerous unit operations. Adding lutein and zeaxanthin to the product portfolio by way of distiller’s grains would add another, high-value stream exiting bioethanol facilities; however, processing conditions throughout production also may affect lutein and zeaxanthin content in the remaining DDGS due to their oxidative potential and thermolability. While more research is needed to specifically analyze and trace carotenoids throughout the refining process, lutein and zeaxanthin are most concentrated in the wet cake or CDS (or both). Studies have shown the presence of crude corn oil in both CDS and the DG [9], indicating that lutein and zeaxanthin likely end up in both fractions after centrifugation; some carotenoids may linger within solid fractions of the remnant kernels. CDS can be further fractioned to produce a more concentrated corn oil that is particularly rich in carotenoids, in some cases more so than DDGS [10]. DDGS can be sold with or without this corn oil, resulting in different fat contents and feed properties of the resulting grain [1]. It is well established that a significant fraction of lutein and zeaxanthin partitions into distillers’ oil during processing, and this oil is often brightly colored due to its carotenoid content. Quantitative partitioning varies by facility and process, but the oil fraction should be considered in future mass balance and recovery studies.

Lutein and zeaxanthin have been shown to partition in primarily the corn endosperm during kernel development [11–14], specifically within amyloplasts [15]. Amyloplasts are the starch-storage sites of plants and are typically low in carotenoid content, but amyloplasts in corn contain substantial amounts of lutein and zeaxanthin within their membranes [16, 17]. Conversion of starch to sugar in the liquefaction step of refining breaks down the remaining contents of amyloplasts [16] via α-amylase and glucoamylase, as well as other enzymes [1, 18]. The post-fermentation whole stillage thus contains remnants of the fractionated corn endosperm, which may account for its presence in both CDS and DDGS.

The question regarding how specific unit operations alter the carotenoid content of corn processing co-products remains. There is a growth in the demand for lutein and zeaxanthin for the United States and global nutraceutical market. Lutein and zeaxanthin contribute to the retinal macular pigment’s formation and result in photoprotective properties that safeguard against age-related macular degeneration [19]. The increasing awareness of the intricacies of proper nutrition and a large aging population are driving the nutraceutical market’s growth; the market is forecasted to grow at a compounded annual growth rate of 6.4% from 2024 to 2029 and is projected to reach a worth of $571.3 billion globally by the end of the forecast period [20]. Carotenoids specifically are also expected to contribute to a growing market. In 2027, the global market for carotenoids is expected to be valued at $2.7 billion [21]. While much research has been conducted on the importance of carotenoids for human and livestock health, processing techniques have remained fundamentally unchanged [21]. Carotenoids tend to be derived via solvent extractions or chemical synthesis, as is the case with lutein and zeaxanthin [21, 22]. Synthetic routes for production have been developed but are cost prohibitive compared to the traditional extraction of lutein and zeaxanthin from marigold petals, the current primary industrial source of these carotenoids [22, 23]. Existing extraction techniques are time consuming and produce large quantities of toxic byproducts. There thus exists a need for manufacturing techniques that are more sustainable and less capital intensive.

DDGS (among other commercial corn products) has been shown in previous studies to be a source of lutein and zeaxanthin that repurposes a byproduct stream to yield value-added products [5, 24]. While Soxhlet extraction is not generally considered a scalable extraction technique, its position as a standard for extracting biomass components lends itself well to understanding the potential of DDGS as a carotenoid source. Soxhlet extraction was employed in this study solely as a standardized laboratory method for quantifying carotenoid content in DDGS samples. The results are intended to inform future assessments of scale-up potential and economic viability, rather than to propose Soxhlet extraction as an industrial process. Given the number of variables that can contribute to lutein and zeaxanthin content in corn kernels and subsequent processing products, the variability in DDGS may have an impact on its viability as a reliable commercial source of lutein and zeaxanthin; feedstock variability affects yield, which affects profit. The present work aims to investigate the extent to which DDGS obtained from five distinct bioethanol facilities yield lutein and zeaxanthin. Particle size is briefly examined to illustrate any processing discrepancies using the milling unit operation as an example. Statistical analysis is employed to draw inferences on carotenoid yield and how different facilities create similar or contrasting DDGS.

## Materials and methods

### Materials

Defatted DDGS samples from five Indiana bioethanol facilities were acquired and partitioned into freezer bags to be stored at -9 °C until needed. 200 proof ethanol (Deacon Labs) was used for extraction, and 99.99% purity nitrogen from Indiana Oxygen was used to dry extraction resins. Methanol and ethyl acetate (HPLC grade, Fisher Scientific) were used for resin reconstitution for HPLC analysis. Gradient RP-HPLC (Waters e2695) was used for analysis, with mobile phase A consisting of HPLC grade methanol (Fisher Scientific), > 98% ammonium Acetate (Fisher Scientific), and ACS grade glacial acetic acid (Fisher Scientific). Mobile phase B was HPLC grade ethyl acetate (Fisher Scientific). Lutein and zeaxanthin standards (Sigma-Aldrich) were diluted in HPLC grade methanol in a 0.01% (w/v) > 99% butylated hydroxytoluene solution (Sigma–Aldrich) and were stored at –25 °C.

### Moisture content analysis

Initial moisture content was collected in triplicate for each sample of grains upon receipt. Moisture content was measured using a Mettler-Toledo HB43 Halogen Moisture Analyzer. Samples were randomly selected samples from within each grain batch and reported as percent solids. Samples were considered dry when there was less than < 1 mg of change in mass in 50 s when dried at 105 °C. The average percent solids were taken to represent the solids content of the entire grain batch and used to inform the loading of samples for each extraction. Moisture content was measured for each DDGS sample and used to adjust biomass loading for extraction. However, solvent volume was not further adjusted to account for differences in sample moisture content, which may have resulted in solvent dilution effects.

### Particle size

Each sample was analyzed for particle size distribution based on ASAE S319.5, with modifications. A set of US Sieve No. 16, 25, 100, and 230 (with a pan) were given an initial charge of around 10 g of grains. Grains were shaken vigorously in 5-min increments until the mass present on the smallest sieve/pan changed by less than 0.1% of the initial charge (around 0.01 g of change). The geometric diameter of the particles was determined using the equations defined in ASAE S319.5 [25].

### Soxhlet extraction

Soxhlet extraction was performed based on [26] and [5]. Extractions were performed with ethanol on all received samples in a randomized order. True replicates were collected in groups of four. The amount of dry mass was kept constant by loading all extractions at 30 mL of solvent per gram of dry sample measured using a Mettler-Toledo (PB1502-S) balance and calculated using the moisture content of each sample. Extraction was maintained at a reflux rate of 6–10 cycles per hour for a duration of 4 h. Extracts were dried at 65 °C (QuikVap Evaporating System, Environmental Express) under nitrogen before drying for 24 h in a 1415 M VWR vacuum oven at 42 °C. For HPLC analysis, resins were reconstituted in a known volume of 50:50 methanol/ethyl acetate solution and filtered using 0.45 µm nylon filters. Chromatographic peaks were related to concentration via calibration curves, and total extractive mass was calculated using the known reconstitution volume.

### HPLC analysis

HPLC analysis was based on [5]. A two-solvent system was used in sample separation and analysis; solvent A consisted of 98:2 methanol/ammonium acetate buffer (1 M ammonium acetate in water adjusted to pH 4.6 using acetic acid), and solvent B was 100% ethyl acetate. Samples were analyzed using a Waters e2695 Autosampler with linear gradient elution:0–80% B for 0–10 min, 80–100% B for 10–12 min, 100% B for 12–14 min, 100–0% B for 14–24 min, and 0% B for 24–30 min. Separation was conducted using a YMC C30 Carotenoid column (150 × 2.0 mm I.D. s-3 µm) with a matching guard column. Signal was recorded using a Waters 2489 UV detector set to 445 nm for lutein and 450 nm for zeaxanthin. Calibrations were performed using analytical standards stored in 100% methanol with 0.01% (w/v) butylated hydroxytoluene as an antioxidant for standard longevity. Waters integration and processing software on Empower 3 was used to identify and quantify peaks, with manual inspection to confirm.

### Statistical design

The Kruskal–Wallis test was employed to determine if facility of origin caused a significant difference in extractive yield for both lutein and zeaxanthin. Samples were randomly assigned an extraction order. An interpretive linear model was developed and analyzed using SAS 9.4. A significance level of α = 0.05 was used for all tests. The number of replicates per facility was limited by sample availability, and the inherent heterogeneity of DDGS contributed to substantial variability in the dataset. These factors, along with the thermolabile nature of carotenoids, may have affected the robustness of statistical analyses and the comparability of results with previous literature. While the present study provides initial insights into carotenoid variability in DDGS, the experimental design was constrained by practical considerations. Future work should incorporate larger sample sizes, increased replication, and standardized laboratory protocols to improve statistical power and data quality.

## Results and discussion

**Fig. 1:**
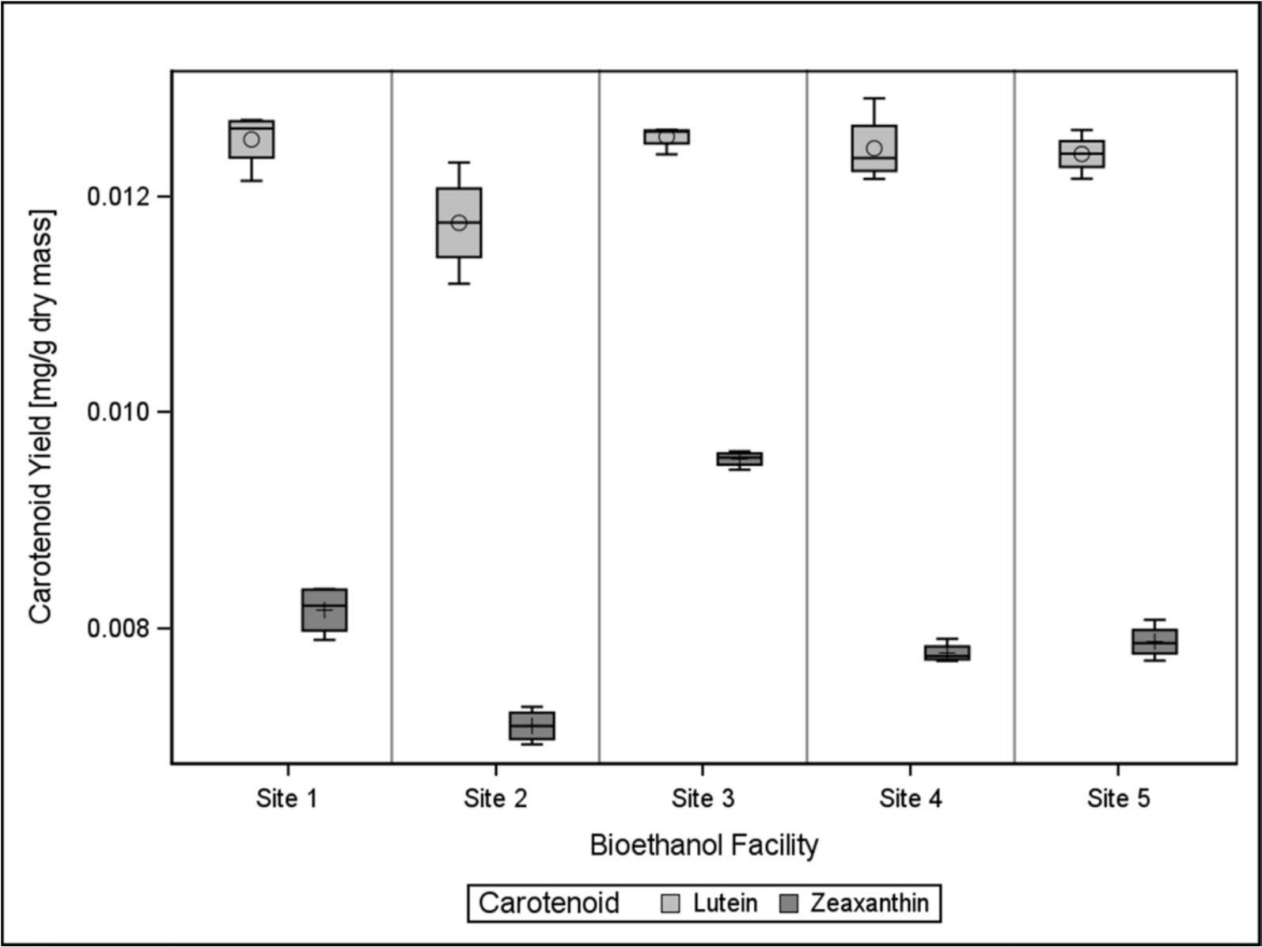
Distributions of lutein and zeaxanthin for each tested Midwestern bioethanol facility. Lutein content remained higher than zeaxanthin content for all facilities but exhibited wider distributions and overlap when compared to zeaxanthin

The distributions of lutein and zeaxanthin can be seen in Fig. 1. Lutein and zeaxanthin are displayed together to illustrate the degree of difference in yield both between carotenoids for a single facility and between facilities for both carotenoids. Lutein yield distributions generally exhibited a greater degree of spread than zeaxanthin. Zeaxanthin distributions, by contrast, appear starkly separated between facilities with a lower degree of variability overall. Reports indicate larger deviations in lutein content than zeaxanthin, while other sources suggest the opposite indicate different levels of deviation in lutein and zeaxanthin content, but overall tending to fluctuate around similar magnitudes overall [5, 15, 24, 27, 28]. Thus, it is more likely that differences in the spread of lutein versus zeaxanthin content may be attributed to the biomass used and laboratory methods rather than a universal underlying phenomenon.

Statistically, the distributions for each bioethanol facility were analyzed first using conventional ANOVA. Initially, analyses for both carotenoids indicated that facility is a significant factor in determining yield; however, diagnostic plots were examined to confirm the validity of ANOVA for the dataset. While normal probability plots confirmed the normality of the dataset, scale-location plots, which chart each residual against its expected value, indicated that variance was not homogeneous for neither lutein nor zeaxanthin yield. It was, therefore, determined that the nonparametric version of ANOVA, the Kruskal–Wallis test, would be used to analyze yield. The Kruskal–Wallis test is an extension of the nonparametric form of the t-test (the Wilcoxon rank sum test) extended to multiple samples.

Both methods retained a significance level of α = 0.05, but upon nonparametric analysis, it was revealed that the differences in lutein yield were no longer significant; distributions of lutein for each facility were statistically identical (χ_4_^2^ = 0.09 > α). Zeaxanthin yield remained significantly impacted by facility (χ_4_^2^ = 0.002 > α). Regarding the distributions of lutein for each facility in Fig. 1, this is not surprising. Lutein yield shows a greater degree of variability than zeaxanthin, with a considerable amount of overlap between site distributions. Visual inspection alone indicates that differences in lutein yield, if any, are small. The lack of significance may indicate a need for more replicates, or a standard lutein yield to be expected from DDGS regardless of facility, as long as the same treatment is received. Given the significance of facility with regard to zeaxanthin, however, it is likely that facility does play at least some part in determining carotenoid yield. Isomerization and degradation of samples during processing also must be considered. HPLC calibration was performed with well-maintained standards, but lutein and zeaxanthin are thermolabile and prone to oxidation and may isomerize in the presence of certain conditions [29]. These factors have the potential to alter molecular structures to the point of splitting peaks on a chromatogram, especially when a C30 column is used.

**Fig. 2:**
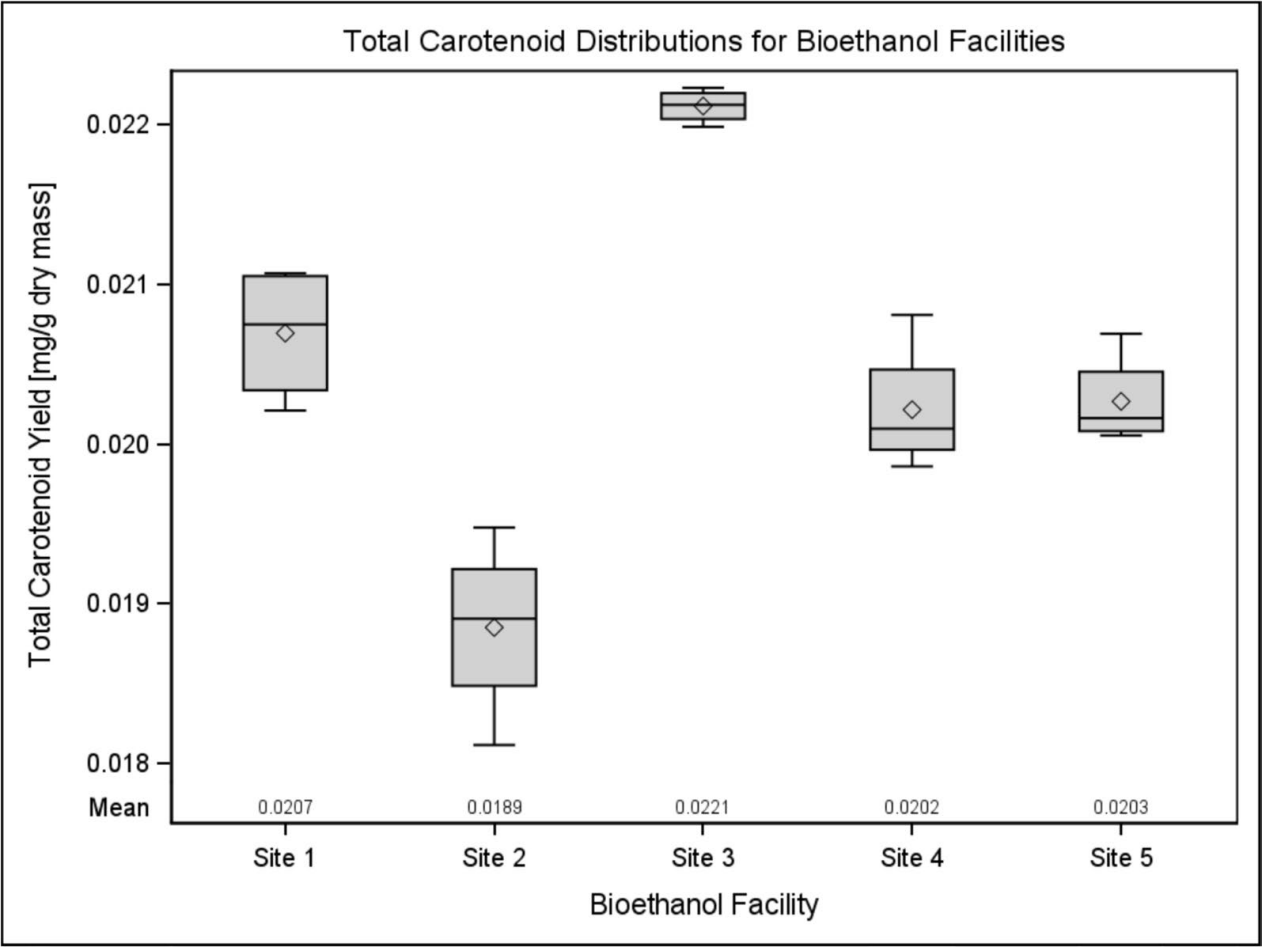
Cumulative carotenoid content for each facility. Even when lutein and zeaxanthin were combined and analyzed together, carotenoid yields were found to be statistically different for each facility

Because lutein and zeaxanthin are sold together in nutritional supplements, an additional analysis was conducted on the cumulative carotenoid yield for each facility (Fig. 2). Although lutein displayed statistically consistent yield while zeaxanthin did not, the variability of zeaxanthin content in DDGS may be ignored if the overall carotenoid content is stable. Since variance was found again to be nonhomogeneous, the Kruskal–Wallis test was used, and it was again determined that the variability between facilities was statistically significant. In spite of carotenoid yields being compiled into a single distribution for each facility, the differences did not stabilize enough to balance out yield differences. Even when combined, carotenoid variability is significant (χ_4_^2^ = 0.003 > α), implying DDGS as a feedstock is likely to encounter difficulties in maintaining consistent supply.

**Fig. 3:**
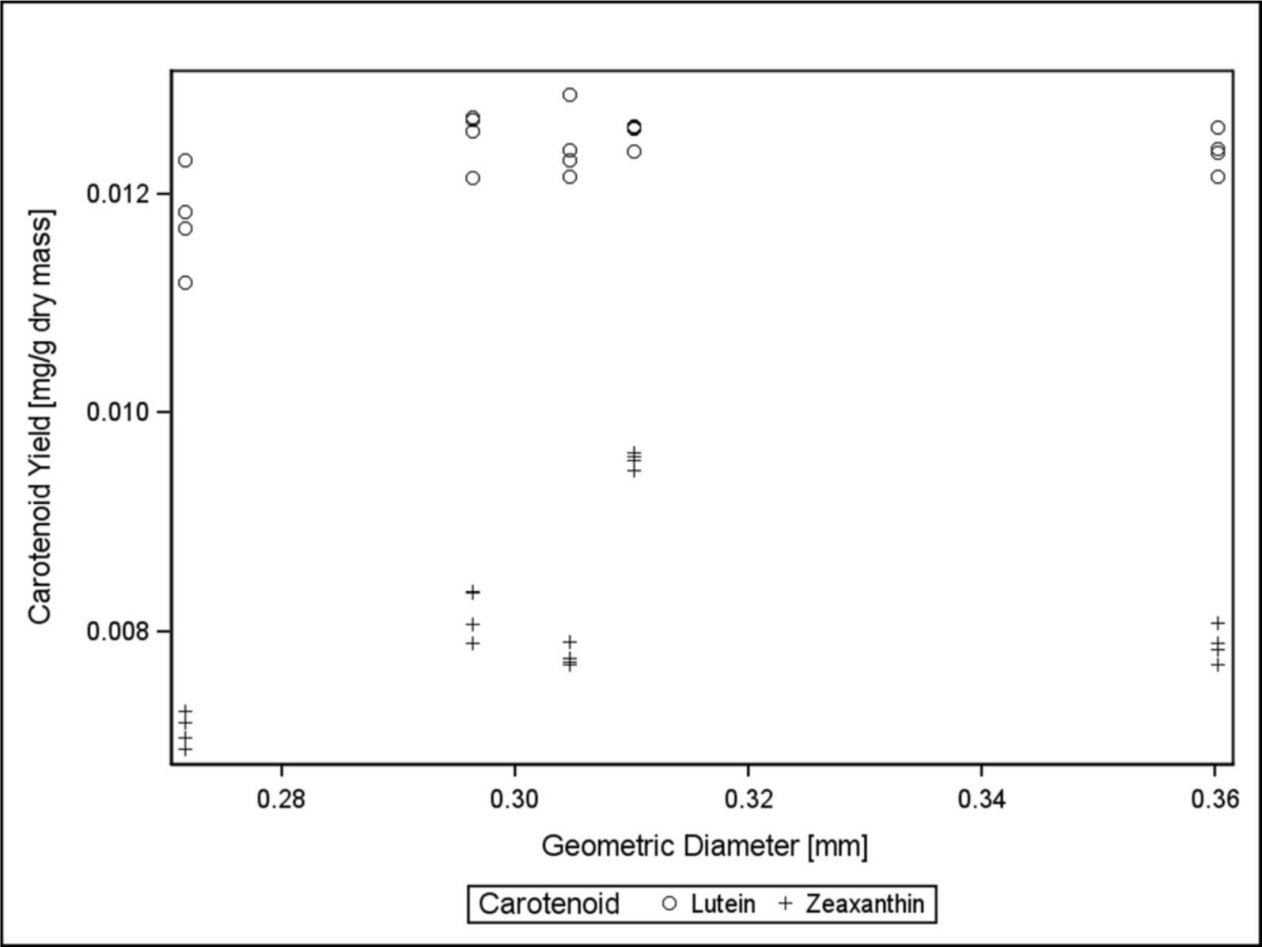
Carotenoid yields plotted as a function of geometric particle sizes, to determine from preliminary data if any trends might be visible. Only one replicate of particle size data was collected to ascertain if any correlations were immediately obvious. No trends or correlations are strongly present in the data; there is the indication of a slight maximum in the center of the chart, but this motif is not strongly indicated in lutein yield

Particle sizes were given a cursory examination to provide numerical characterization to visual differences present between received samples. Geometric diameters and standard deviations of samples are listed in Table 1. No correlations were observed between diameter and lutein and zeaxanthin yield (Fig. 3). The potential impact of sample moisture content on extraction efficiency and carotenoid yield represents a limitation of this study. Future research should investigate whether a correlation exists between moisture content and carotenoid recovery, and adjust solvent volumes accordingly. An inability to uncover any correlations that might link particle size to yields indicated that particle size, while potentially an important factor, was likely not linked to the processing operation that also causes dramatic differences in carotenoid yield. Statistical significance in geometric diameter was considered; however, as noted, no strong trends or correlations between geometric diameter and carotenoid yield were observed. Therefore, further statistical analysis of particle size was not pursued in this study. That is, particle size differs as a result of processing in the same way carotenoid yield might; both could be considered dependent variables and might be related but are only useful in identifying linchpin operations in the bioethanol facility—not in describing causative trends in relation to each other.

**Table 1:**
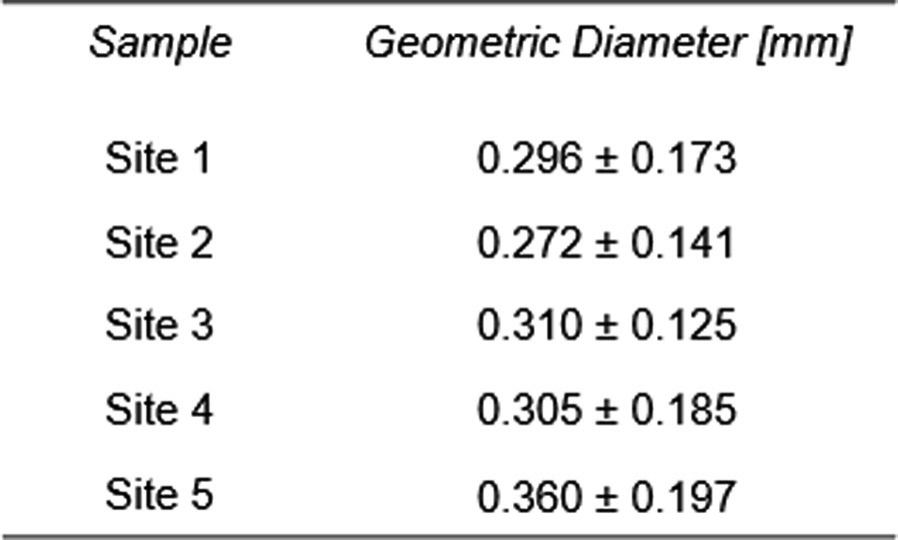
Approximate particle sizes of samples received reported by their geometric diameters and corresponding geometric standard deviations

The contrasting significance for lutein and zeaxanthin illustrates the need to further investigate factors affecting carotenoid content in DDGS. In terms of feedstock reliability for commercial-scale extraction, uniformity between facilities is sought; less differences between facilities indicates a level of standardization better suited for byproduct use. The potential impact of sample moisture content on extraction efficiency and carotenoid yield represents a limitation of this study. Future research should investigate whether a correlation exists between moisture content and carotenoid recovery, and adjust solvent volumes accordingly. Only lutein yields for each facility could be attributed to the same overall distribution in terms of statistical analysis, zeaxanthin yield displayed significant differences. Explaining this effect with any one factor would be a fallacy. Upstream to DDGS production, grain undergoes a series of processing steps in preparation for ethanol extraction and distillation, and further processing is required to turn whole stillage into the final dried grain. Each of these steps, if not done with precise control and replicability, has the potential to affect grain quality and therefore lutein and zeaxanthin content. Prior to processing, corn on the field is subject to a dizzying number of conditions that can affect lutein and zeaxanthin content. Differences in rainfall, geography, and hybrid type could be considered factors that might affect lutein and zeaxanthin within the kernel, as well as bioethanol yield as a whole [15, 30–32]. Not all steps will likely bear the same impact. As with bioethanol processing steps, it is unlikely that all minutiae may cause significant differences in carotenoid content; however, these factors remain uncontrolled and therefore their contributions are not to be overlooked.

## Conclusions

Bioethanol facilities do not follow standard procedures for producing DDGS. While the overarching process remains the same, mixing ratios of CDS and WDGS, backset streams, and corn oil extraction procedures vary, contributing to differences in carotenoid yields. The five tested Midwestern facilities displayed significant differences in zeaxanthin yield, while lutein yield showed broader variance and was not facility-specific. The experimental design was limited by sample size, replication, and the thermolabile nature of carotenoids, resulting in substantial dataset variability and reduced statistical robustness. The potential impact of sample moisture content on extraction efficiency and carotenoid yield represents a limitation; future research should investigate this relationship and adjust solvent volumes accordingly. While further economic analysis is required, the results suggest that DDGS from corn ethanol facilities could represent a materially significant source of lutein and zeaxanthin, warranting investigation into scalable recovery and market viability. Future work should include longitudinal sampling at a single facility, incorporating unprocessed corn, DDGS, and distillers’ oil, to establish a rudimentary mass balance for carotenoid recovery. Overall, this preliminary investigation provides a foundation for more comprehensive studies to fully assess the feasibility of DDGS as a feedstock for lutein and zeaxanthin production.

## Data Availability

The datasets used and/or analyzed during the current study are available from the corresponding author on reasonable request.

## Abbreviations

ANOVA: Analysis of variance
CDS: Condensed distiller’s solubles
DDGS: Dried distiller’s grains and solubles
DG: Distiller’s grains
HPLC: High-performance liquid chromatography
WDG: Wet distiller’s grains
WDGS: Wet distiller’s grains and solubles
